# Bilateral Simultaneous Acute Angle‐Closure Glaucoma Following Over‐the‐Counter Cold Medication

**DOI:** 10.1155/crop/8847063

**Published:** 2026-02-27

**Authors:** Chiara Vivarelli, Marco Pellegrini, Francesco Parmeggiani, Ginevra Giovanna Adamo, Carla Enrica Gallenga, Paola Ferri, Marco Mura

**Affiliations:** ^1^ Ophthalmology Clinic Operating Unit, Head and Neck Department, Arcispedale Sant′Anna University Hospital, Ferrara, Italy; ^2^ Department of Translational Medicine, University of Ferrara, Ferrara, Italy, unife.it; ^3^ Department of Ophthalmology, Ospedali Privati Forlì “Villa Igea”, Forlì, Italy; ^4^ Department of Biomedical Metabolic and Neural Sciences, University of Modena and Reggio Emilia, Modena, Italy, unimore.it; ^5^ Department of Vitreoretinal and Uveitis Divisions, King Khaled Eye Specialist Hospital, Riyadh, Saudi Arabia, kkesh.med.sa

**Keywords:** bilateral acute angle-closure glaucoma, case report, drug-induced acute angle-closure glaucoma, mydriasis, over-the-counter flu medication

## Abstract

**Purpose:**

We report a case of bilateral acute angle‐closure glaucoma occurring concurrently after the intake of dextromethorphan, a commonly used over‐the‐counter medication for flu symptom relief.

**Case Presentation:**

A 65‐year‐old woman presented with bilateral acute angle‐closure glaucoma following 5 days of treatment with an OTC medication. Her intraocular pressures (IOPs) were 57 mmHg in the right eye and 50 mmHg in the left. Slit lamp biomicroscopy revealed shallow anterior chambers bilaterally, and gonioscopy confirmed angle closure. Management involved immediate discontinuation of the offending medication, initiation of antiglaucoma treatment, and bilateral Nd: YAG laser iridotomy, which successfully normalized IOP and resolved the angle‐closure attack.

**Conclusion:**

This case highlights that commonly used OTC cold and flu remedies can trigger acute angle‐closure glaucoma, particularly in predisposed individuals. Early diagnosis and prompt intervention are crucial to prevent vision loss.

## 1. Introduction

Bilateral simultaneous acute angle‐closure glaucoma (AACG) is a rare but serious event that often occurs in predisposed eyes, such as those with short axial length, a thick crystalline lens, narrow angles, or a shallow anterior chamber [1]. Many cases are precipitated by pharmacological agents that induce mydriasis and cycloplegia.

A wide range of medications can act as triggers for drug‐induced AACG, including *α*‐adrenergic and *β*2‐adrenergic agonists, anticholinergic and cholinergic agents, H1 and H2 receptor antagonists, selective serotonin reuptake inhibitors, and tricyclic and tetracyclic antidepressants. In addition, benzodiazepines, bronchodilators, vasoconstrictors, and anti‐Parkinson agents can precipitate angle closure in susceptible individuals. Another mechanism that can cause AACG involves anterior displacement of the iris‐lens diaphragm, commonly associated with sulfa‐based drugs such as topiramate.

We report a case of bilateral simultaneous AACG in a patient following multiple doses of an over‐the‐counter cold and flu remedy containing paracetamol, dextromethorphan, and guaifenesin, for which no warning regarding ocular side effects was indicated on the product labeling.

## 2. Case Presentation

A 65‐year‐old woman presented to the emergency department of Sant’Anna University Hospital (Ferrara, Italy) with a 2‐day history of photophobia, bilateral blurry vision and exhibited fixed, mid‐dilated pupils. She also reported general malaise, nausea, vomiting, and frontal headache. She had no prior history of similar episodes.

Her medical history was unremarkable, except for a recent upper respiratory tract infection with fever that began 5 days earlier. She reported taking an over‐the‐counter flu remedy containing paracetamol, dextromethorphan, and guaifenesin for the past 5 days and denied using any other medications. She had no significant ocular history, aside from hyperopia (+ 3.00 diopters spherical equivalent “oculus uterque”), nor any family history of ocular disease.

On examination, her pulse rate was 72 bpm and her blood pressure was 128/84 mmHg. The patient was initially assessed by a primary care physician, who suspected a central neurological disease. Brain computed tomography scan, neurological evaluation, infectious disease consultation, and lumbar puncture were performed, all yielding unremarkable results. She was subsequently referred to the ophthalmology emergency service.

Upon admission to the ophthalmology unit, her best visual acuity was limited to light perception in both eyes. Slit‐lamp biomicroscopy showed conjunctival–ciliary mixed injection, severe corneal stromal edema with endothelial folds, shallow anterior chamber with no evidence of iris bombe, nuclear cataracts, and mid‐dilated pupils in both eyes (Figure 1a,b).

Figure 1(a) Right eye and (b) left eye: color photographs showing hyperemic conjunctiva, corneal edema with endothelial folds, shallow anterior chamber, cataract, and a mid‐dilated pupil.

**Figure:**
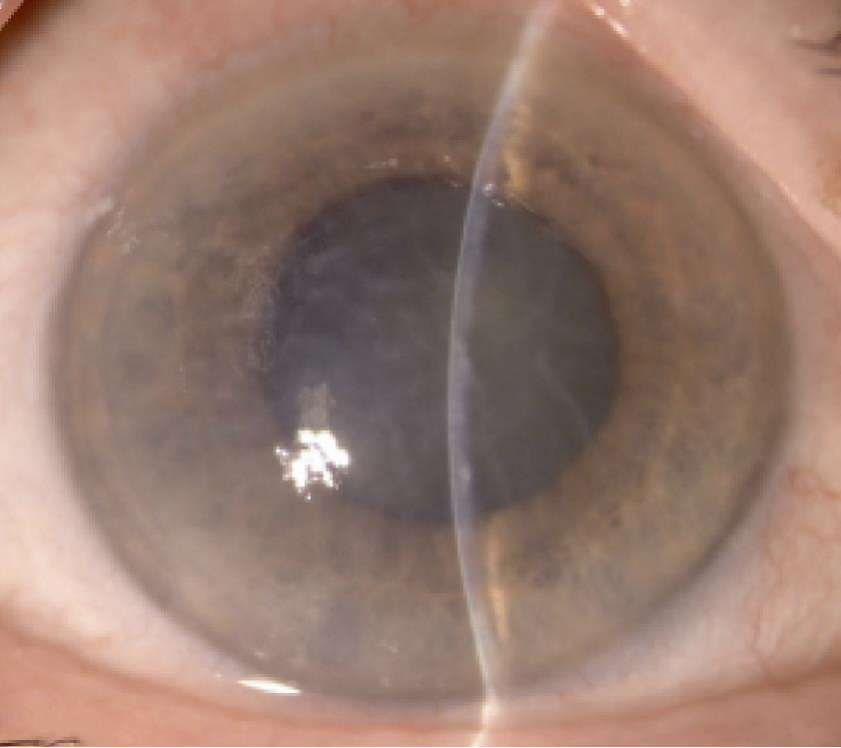
(a)

**Figure:**
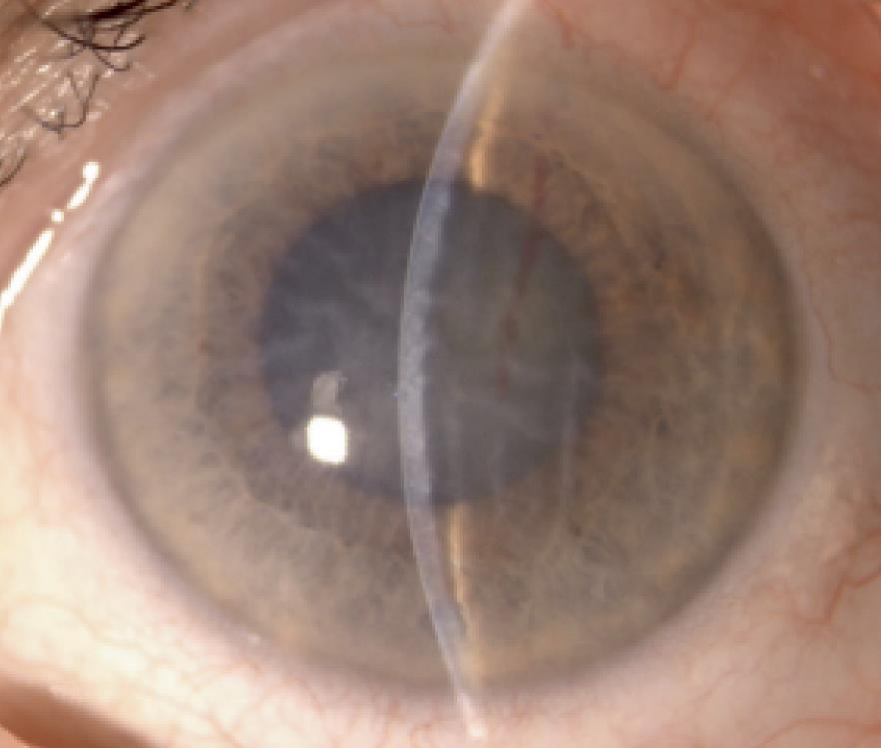
(b)

IOP, measured with Goldmann applanation tonometry, was 57 mmHg in the right eye and 50 mmHg in the left eye. Gonioscopy revealed complete angle closure without peripheral anterior synechiae in the four quadrants of both eyes. Anterior segment optical coherence tomography (AS‐OCT) confirmed narrow angles bilaterally (Figure 2a,b). A diagnosis of simultaneous bilateral AACG was made.

Figure 2(a) Right eye and (b) left eye: anterior segment optical coherence tomography following the resolution of angle closure showing a shallow anterior chamber and a narrow irido‐corneal angle. The images also show corneal edema with endothelial folds and nuclear cataract with dense central opacity. The lens is disproportionately large within a short hyperopic axial length.

**Figure:**
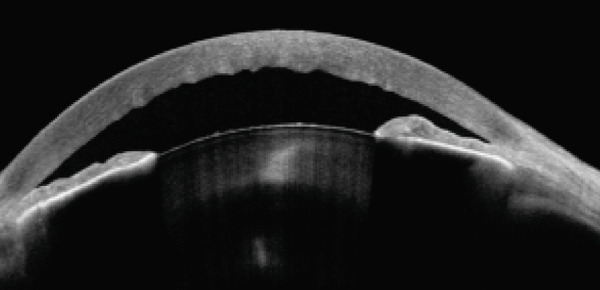
(a)

**Figure:**
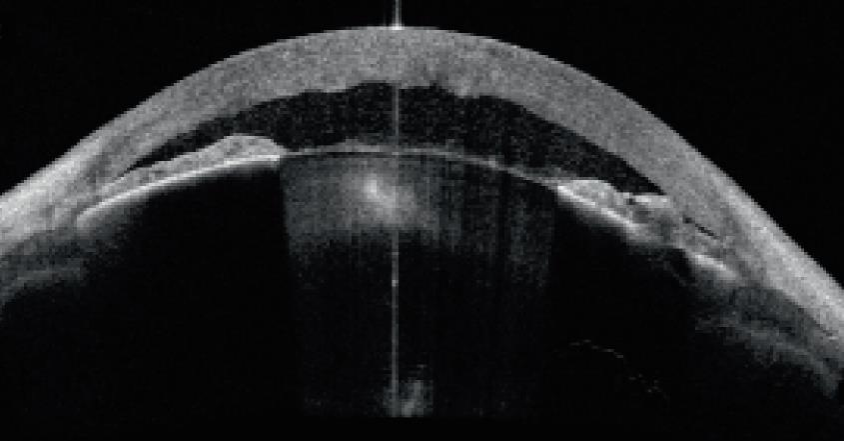
(b)

**Figure 3:**
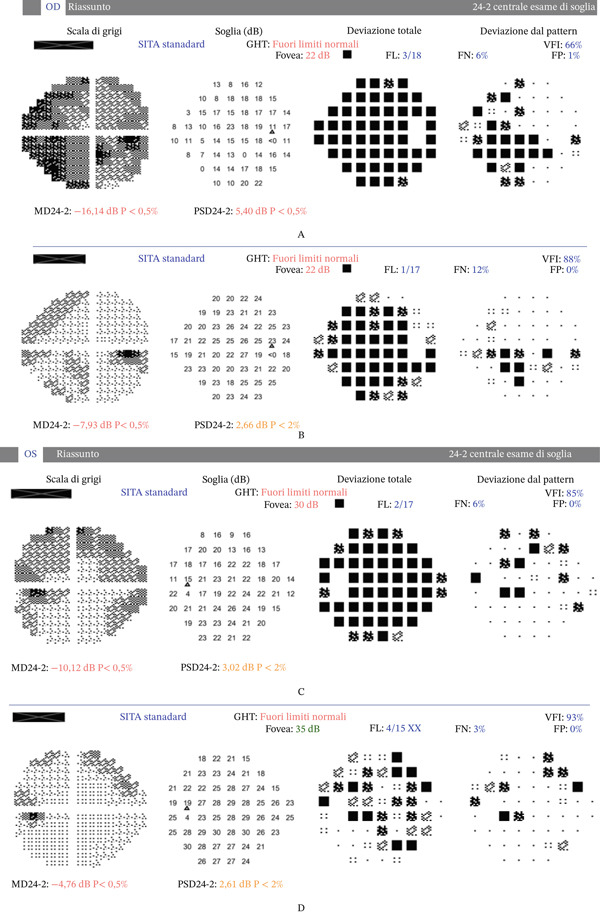
(A) Humprey visual perimetry of the right eye performed before phacoemulsification showing severe generalized depression with reduced foveal sensitivity (22 dB). The defect is both diffuse and localized. (B) Humprey visual perimetry of the left eye performed before phacoemulsification showing generalized depression with a foveal threshold of 30 dB. (C) Humprey visual perimetry of the right eye performed after phacoemulsification showing marked improvement in overall sensitivity with a foveal sensitivity of 27 dB and a paracentral inferonasal arcuate defect, suggesting the presence of functional glaucomatous damage. (D) Humprey visual perimetry of the left eye performed after phacoemulsification showing marked improvement in overall field with a foveal sensitivity improved to 35 dB.

The flu medication was immediately discontinued, and the patient was started on systemic and topical antiglaucoma therapy, including intravenous mannitol 18% 250 mL, topical timolol 0.5%, pilocarpine 2%, and brimonidine 0.1%, oral acetazolamide 250 mg three times daily. The following day, corneal edema had improved sufficiently to allow Nd:YAG laser peripheral iridotomies in both eyes, which resolved the pupillary block and normalized the IOP. At that time, visual acuity improved slightly to hand motion in both eyes. Phacoemulsification surgery was performed in both eyes, leading to a final best corrected visual acuity of 20/32 in the right eye and 20/20 in the left eye. The right optic nerve exhibited greater cupping on ophthalmoscopy. Automated perimetry revealed a paracentral inferonasal arcuate scotoma in the right visual field (Figures 3a, 3b, 3c, and 3d). At the 6‐month follow‐up visit, the patient reported a mild reduction in visual acuity. Optical coherence tomography demonstrated bilateral cystoid macular edema, consistent with Irvine–Gass syndrome. Treatment with topical nonsteroidal anti‐inflammatory drugs resulted in resolution of the macular edema and recovery of visual acuity.

**Figure 4:**
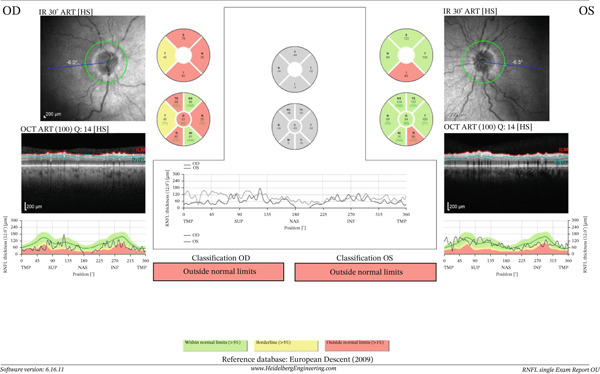
OCT scan (RNFL) showing more diffuse and severe loss in the right eye in comparison to the left eye.

**Figure 5:**
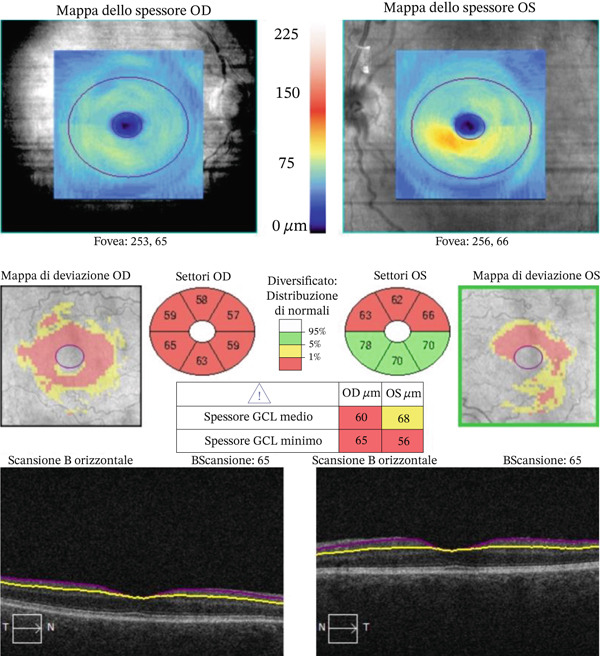
OCT scan (GCIPL) showing thinning of the ganglion cell layer in both eyes, in particular in the right eye.

Optical coherence tomography of retinal nerve fiber layer (RNFL) and ganglion cell‐inner plexiform layer (GCIPL) showed greater structural damage in the right eye, supporting the finding of more advanced cupping (Figures 4 and 5).

## 3. Discussion

Several over‐the‐counter cold and flu medications have been implicated in the precipitation of AACG, particularly in individuals with predisposing ocular anatomy [2–7]. In this case, bilateral simultaneous AACG was likely induced by a common remedy containing dextromethorphan, which is not commonly recognized as an ocular risk factor. To date, two case reports have described AACG following the use of dextromethorphan‐containing medications [2, 8]. One report described a case of unilateral AACG triggered by a cold remedy containing dextromethorphan, promethazine, and phenylpropanolamine [2]. However, in that case, both promethazine (an antihistamine with antimuscarinic effects) and phenylpropanolamine (a sympathomimetic agent) may have contributed to mydriasis, thereby precipitating AACG. More recently, a case of bilateral AACG associated with dextromethorphan use has been reported [8]. Conversely, in our patient, since neither paracetamol nor guaifenesin has sympathomimetic or anticholinergic effects, dextromethorphan was likely the sole precipitating factor. Dextromethorphan is a synthetic derivative of morphine and is structurally related to opioids but does not activate mu‐opioid receptors. At high doses, dextromethorphan and its active metabolite, dextrorphan, act as N‐methyl‐D‐aspartate receptor antagonists, leading to central nervous system stimulation. In addition, norepinephrine reuptake is inhibited [9]. Ultimately, these mechanisms lead to an increased concentration of peripheral norepinephrine, which can stimulate *α*1‐adrenergic receptors located on the radial muscle of the iris. In individuals with narrow anterior chamber angles, mid‐dilation is the most dangerous state, as the lens moves closer to the iris, potentially creating a pupillary block [10]. Concurrently, contraction of the iris dilator muscle causes the iris to thicken peripherally, which can lead to crowding of the iridocorneal angle. Furthermore, dextromethorphan acts as an agonist at Sigma‐1 receptors, which modulate multiple neurotransmitter systems, including dopamine and norepinephrine [9]. Another potential pathophysiological mechanism occurs when, at high doses, dextromethorphan acts as a weak serotonin reuptake inhibitor, raising extracellular serotonin levels [9]. Notably, 5‐HT7 receptors are expressed in the iris sphincter muscle as well as in the pigmented and nonpigmented epithelial layers of the pars plicata region of the ciliary processes. Stimulation of 5‐HT7 receptors can lead to relaxation of the iris sphincter muscle and an increase in aqueous humor production. Moreover, 5‐HT2A and 5‐HT2C receptors, also located in the iris‐ciliary body complex, are associated with increased ciliary body blood flow, further enhancing aqueous humor production [11–16].

Another key factor contributing to the bilateral attack in this scenario was that the patient was hyperopic [1]. Finally, another possible contributing mechanism may be the presence of fever and systemic illness, which can stimulate the sympathetic nervous system, leading to an increase in the release of peripheral norepinephrine. This case underscores the importance of including bilateral AACG in the differential diagnosis in patients presenting with photophobia, bilateral blurry vision, fixed mid‐dilated pupils, and systemic symptoms such as nausea, vomiting, and headache. Although these signs may initially suggest neurological or infectious conditions, failing to consider an ocular etiology can result in delayed diagnosis and treatment. Timely recognition of angle‐closure mechanisms is critical to prevent permanent vision loss and improve patient outcomes. This report highlights that over‐the‐counter cold and flu medications containing dextromethorphan can trigger AACG in susceptible individuals. Healthcare providers, particularly those in acute care settings, must remain vigilant for this potential drug‐induced complication. Moreover, pharmacists should be aware of the ocular side effects of medications that influence pupil dilation, aqueous humor dynamics, and intraocular pressure.

## 4. Conclusions

In conclusion, this case illustrates that over‐the‐counter medications containing dextromethorphan can trigger bilateral AACG in predisposed individuals. Clinicians and pharmacists should be aware of this risk to ensure prompt diagnosis and prevent vision loss. Additionally, the package leaflet for this OTC remedy must be updated to include the following rare adverse event citation by the Regulatory Authority.

**Nomenclature**
OTC: over‐the‐counter
IOP: intraocular pressure
AACG: acute angle‐closure glaucoma
AS‐OCT: anterior segment optical coherence tomography
RNFL: retinal nerve fiber layer
GCIPL: ganglion cell‐inner plexiform layer

## Funding

Open access publishing facilitated by Università degli Studi di Ferrara, as part of the Wiley ‐ CRUI‐CARE agreement.

## Disclosure

All authors attest that they meet the current ICMJE criteria for authorship.

## Ethics Statement

All procedures were in accordance with the ethical standards of the Helsinki Declaration 1964 and later versions. Consent to publish this case report has been obtained from the patient in writing.

## Conflicts of Interest

The authors declare no conflicts of interest.

## Data Availability

The data that support the findings of this study are available from the corresponding author upon reasonable request.
